# Vagus Nerve Stimulation Ameliorates Renal Ischemia-Reperfusion Injury through Inhibiting NF-*κ*B Activation and iNOS Protein Expression

**DOI:** 10.1155/2020/7106525

**Published:** 2020-02-20

**Authors:** Meng Wang, Jielin Deng, Huanzhu Lai, Yanqiu Lai, Guannan Meng, Zhenya Wang, Zhen Zhou, Hu Chen, Zhiyao Yu, Shuyan Li, Hong Jiang

**Affiliations:** ^1^Department of Cardiology, Renmin Hospital of Wuhan University, Wuhan, 430060 Hubei, China; ^2^Cardiovascular Research Institute, Wuhan University, Wuhan, 430060 Hubei, China; ^3^Hubei Key Laboratory of Cardiology, Wuhan, 430060 Hubei, China; ^4^Department of Cardiology, First Hospital of Jilin University, Changchun, 130021 Jilin, China

## Abstract

**Objective:**

In renal ischemia/reperfusion injury (RIRI), nuclear factor *κ*B (NF-*κ*B (NF-*κ*B (NF-

**Methods:**

Eighteen male Sprague-Dawley rats were randomly allocated into the sham group, the I/R group, and the VNS+I/R group, 6 rats per group. An RIRI model was induced by a right nephrectomy and blockade of the left renal pedicle vessels for 45 min. After 6 h of reperfusion, the blood samples and renal samples were collected. The VNS treatment was performed throughout the I/R process in the VNS+I/R group using specific parameters (20 Hz, 0.1 ms in duration, square waves) known to produce a small but reliable bradycardia. Blood was used for evaluation of renal function and inflammatory state. Renal injury was evaluated via TUNEL staining. Renal samples were harvested to evaluate renal oxidative stress, NF-*κ*B (NF-

**Results:**

The VNS treatment reduces serum creatinine (Cr) and blood urea nitrogen (BUN) levels. Simultaneously, the levels of tumor necrosis factor alpha (TNF-*α*), interleukin 6 (IL-6), and interleukin 1-beta (IL-1*β*) were significantly increased in the I/R group, but VNS treatment markedly ameliorated this inflammatory response. Furthermore, the VNS ameliorated oxidant stress and renal injury, indicated by a decrease in 3-nitrotyrosine (3-NT) formation and MDA and MPO levels and an increase in the SOD level compared to that in the I/R group. Finally, the VNS also significantly decreases NF-*κ*B (NF-

**Conclusion:**

Our findings indicate that NF-*κ*B activation increased iNOS expression and promoted RIRI and that VNS treatment attenuated RIRI by inhibiting iNOS expression, oxidative stress, and inflammation via NF-*κ*B inactivation.*κ*B (NF-*κ*B (NF-

## 1. Introduction

Acute kidney injury (AKI) is a severe clinical complication of kidney transplantation, bypass, and sepsis [1]. The mortality rates associated with renal ischemia-reperfusion injury (RIRI) remain high, and patients suffering RIRI increase the risk of developing chronic kidney disease (CKD) and end-stage renal disease (ESRD) [2]. Although the underlying mechanisms that link acute kidney injury (AKI) to CKD in humans remain unclear, animal experiments indicate that cytokine release, oxidative stress, reactive oxygen species (ROS) production, and toxicant buildup play a critical role [3].

Nitric oxide (NO), an endogenous gaseous molecule synthesized by the enzyme nitric oxide synthase, plays an essential role in the immune, cardiovascular, and nervous systems [4]. Previous studies have indicated that overproduction of NO from inducible nitric oxide synthase (iNOS) is detrimental to RIRI. The selective iNOS antagonist GW274150 can significantly attenuate RIRI via antioxidative stress [5]. Nuclear factor-*κ*B (NF-*κ*B), a modulator of gene expression, is involved in the expression of multiple genes such as cytokine release, cell survival, apoptosis, and proliferation [6, 7]. Several studies have shown that activating NF-*κ*B and increasing NO overproduction from iNOS aggravate RIRI [8]. Furthermore, inhibiting NF-*κ*B activation by NF-*κ*B decoy oligodeoxynucleotide reduces iNOS expression and ameliorates renal dysfunction induced by ischemia AKI [9].

Recently, animal studies have shown that vagus nerve stimulation (VNS) generates protective effects in sepsis, endotoxemia, arthritis, and other inflammatory syndromes [10]. In addition, VNS improves multiple organ I/R injury, including injury of the liver, heart, and skeletal muscle via antioxidant and anti-inflammatory mechanisms [11–13]. Furthermore, Our previous clinical study found that noninvasive VNS dramatically improves myocardial I/R injury [14]. However, whether VNS is able to modulate NF-*κ*B activation and iNOS expression in the kidney remains unclear. In the present study, we aimed to systematically investigate whether VNS exerts a protective effect on RIRI via inhibiting NF-*κ*B activation and iNOS expression.

## 2. Materials and Methods

### 2.1. Animal Preparation and Experimental Groups

**Figure 1:**
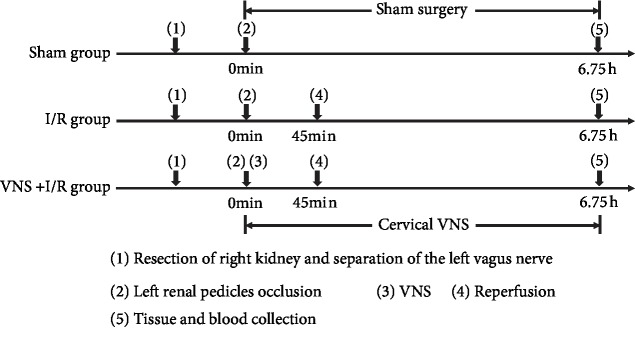
Experimental flowchart of the three groups. I/R: ischemia-reperfusion; VNS: vagus nerve stimulation.

The animal studies were approved by the Experiment Animal Center of Wuhan University, China. The present study was performed according to the Institutional Guidelines for the Care and Use of Laboratory Animals. Eighteen male Sprague-Dawley (SD) rats weighting 250–300 g (8-12 weeks of age) were anesthetized intraperitoneally with Na-pentobarbital (3%, 50 mg/kg). The depth of anesthesia was monitored, and the rectal temperature was maintained at 37°C throughout the experimental procedure. The rats were randomly divided into the control group (sham, *n* = 6), the I/R group (I/R, *n* = 6), or the I/R plus VNS group (I/R+VNS, *n* = 6). After reperfusion, the tissue and blood samples were collected for analysis. The study protocol is presented in Figure 1.

### 2.2. RIRI Model Preparation

The abdominal region was sterilized, and a midline laparotomy was carried out. A right nephrectomy was implemented in each rat. The left renal blood vessels were separated and occluded with a nontraumatic microaneurysm clamp to induce renal ischemia. After 45 min of blockade, the clamp was removed for 6 h of reperfusion as previously described [15]. Renal ischemia-reperfusion was visually confirmed by a change in the color of the renal surface.

### 2.3. VNS Treatment

In this study, the left cervical vagosympathetic trunks of the rats were separated and stimulated (frequency 20 Hz, 0.1 ms duration) through a pair of TeflonR-coated silver wires (0.1 mm in diameter) with a special stimulator (S20, Jinjiang, Chengdu, China). The voltage necessary to achieve a 10% reduction in sinus rate was used as the threshold [11, 16]. The VNS treatment was performed throughout the I/R process.

### 2.4. Assessment of Renal Function and Serum Inflammatory Cytokines

After reperfusion procedure, the blood samples were drawn from the postcaval vein and centrifuged (3,000 rpm, 15 min) at 4°C. The serum creatinine (Cr) and blood urea nitrogen (BUN) levels were measured by an automatic analyzer (Chemray 240 analyzer, Dulei Biotechnology, Shenzhen, China). The serum tumor necrosis factor alpha (TNF-*α*), interleukin 6 (IL-6), and interleukin 1-beta (IL-1*β*) were analyzed by ELISA (ELK Biotechnology) following the manufacturer's instructions.

### 2.5. Assessment of Renal Malondialdehyde (MDA) Level and Myeloperoxidase (MPO) and Superoxide Dismutase (SOD) Activity

Aliquots of renal tissue from the different groups were completely homogenized. Renal MDA, MPO levels, and SOD activity were measured using commercially available chemical assay kits (Nanjing Jiancheng Bioengineering Institute) as instructed by the manufacturer's protocol.

### 2.6. Measurement of Renal Nitrite/Nitrate and 3-Nitrotyrosine (3-NT) Concentrations

As metabolites of NO react with oxygen, the concentration of renal nitrite/nitrate serves as an indicator of NO synthesis. An important reactive nitrogen species (RNS), 3-NT, was used as a biomarker of ONOO^−^ formation during I/R [17]. The levels of nitrite and nitrate were determined via a colorimetric nonenzymatic nitrite/nitrate assay kit (Nanjing Jiancheng Bioengineering Institute). The concentrations of 3-NT in the kidney in each group were determined using a 3-NT assay kit (Elabscience Biotechnology Co., Ltd.).

### 2.7. Western Blot Analysis

Renal iNOS and NF-*κ*B p65 protein level were measured using Western blot analysis. In brief, renal tissue was completely homogenized in buffer. The supernatant was collected to extract total protein. Equal amounts of homogenate protein were separated by SDS-PAGE (ASPEN, Wuhan, China) and then transferred onto a nitrocellulose membrane. The membranes were blocked and incubated with primary antibodies (anti-NOS, Abcam; anti-NF-*κ*B p65, Abcam; and anti-*β*-actin, Abcam) at 4°C overnight. The membranes were washed fully in TBST and incubated with anti-rabbit secondary antibody (Aspen). Finally, the relative protein expression levels were standardized to the level of *β*-actin and quantified using image analyzer software (AlphaEase FC, USA).

### 2.8. Real-Time PCR Analysis

The mRNA expression levels of iNOS were detected by RT-qPCR. Total RNA was extracted from renal tissues with Trizol Reagent (Invitrogen™, Thermo) following the manufacturer's instructions. The RNA was reverse transcribed into first-strand cDNA using a cDNA synthesis kit PrimeScript™ RT reagent kit with gDNA Eraser (TaKaRa Bio Inc.). iNOS mRNA expression was quantified by quantitative real-time PCR using a StepOne™ Real-Time PCR System (Life Technologies). *β*-Actin expression served as the internal control. The iNOS gene expression levels were determined using the 2^−*ΔΔ*CT^ method. The primer sequences were as follows: iNOS, forward: 5′-AGCATCCACGCCAAGAACG-3′, reverse: 5′-GTCTGGTTGCCTGGGAAAAT-3′; mRNA level *β*-actin, forward: 5′-CGTTGACATCCGTAAAGACCTC-3′; reverse: 5′TAGGAGCCAGGGCAGTAATCT-3′.

### 2.9. Histological Staining

After reperfusion procedure, the renal tissues of different groups were fixed with paraformaldehyde at room temperature and then embedded in paraffin. The ischemic renal tissues were cut into serial 4 *μ*m sections and stained with hematoxylin and eosin (H&E) to determine morphological changes in the kidney. The renal tissue images were captured under a light microscope.

### 2.10. Statistical Analysis

All data are presented as the mean ± standard deviation (SD). Between-group differences were detected via one-way analysis of variance (ANOVA). These data were analyzed via GraphPad Prism version 7.0 software (GraphPad Software, Inc., San Diego, CA). *P* < 0.05 indicated a statistically significant.

## 3. Results

### 3.1. Effect of VNS on Renal Function and Tissue Morphology

**Figure 2:**
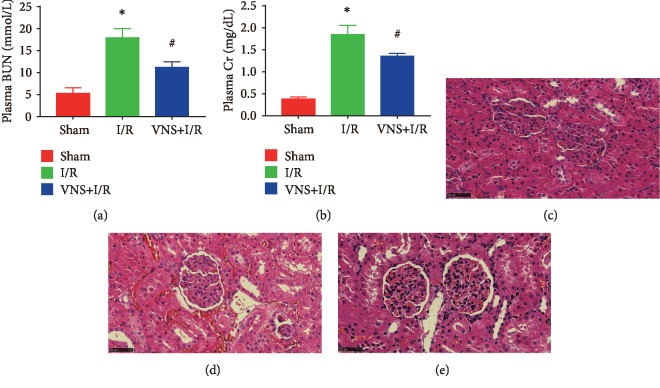
VNS alleviates AKI. (a) The serum concentrations of Cr in three groups. (b) The serum concentrations of BUN in three groups. (c) Representative renal tissue morphology from the kidneys of the sham group. (d) Representative renal tissue morphology from the I/R group. (e) Representative renal tissue morphology from the VNS+I/R group. Data are expressed as mean ± SD with *n* = 6 per group. ^∗^*P* < 0.05 versus the sham group; ^#^*P* < 0.05 versus the I/R group. Cr: creatinine; BUN: blood urea nitrogen.

As shown in Figures 2(a) and 2(b), plasma Cr and BUN levels were determined at the end of I/R. The level of Cr and BUN was markedly higher in the I/R group than that in the sham group (Cr, 0.38 ± 0.05 mg/dL vs. 1.84 ± 0.21 mg/dL, ^∗^*P* < 0.05; BUN, 17.87 ± 2.15 mmol/L vs. 5.28 ± 1.23 mmol/L; ^∗^*P* < 0.05). However, VNS decreased the plasma Cr and BUN levels (1.35 ± 0.07 mg/dL in the I/R+VNS group vs. 1.84 ± 0.21 mg/dL in the I/R group; ^#^*P* < 0.05; BUN, 11.15 ± 1.30 mmol/L vs. 17.87 ± 2.15 mmol/L; ^#^*P* < 0.05), indicating that VNS treatment improved renal function. Furthermore, we examined renal morphology by H&E staining. Consistent with the improvement of renal function, tubular necrosis and interstitial congestion were significantly reduced by VNS treatment (Figures 2(c)–2(e)).

### 3.2. Effect of VNS on Serum Inflammatory Status

**Figure 3:**
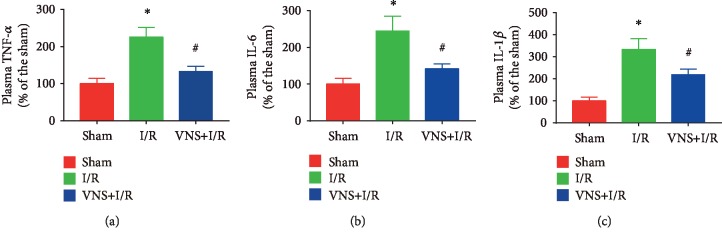
VNS mitigates inflammation in RIRI. (a–c) The effect of VNS on the plasma concentrations of TNF-*α*, IL-6, and IL-1*β* in the different groups. Data are expressed as mean ± SD with *n* = 6 per group. ^∗^*P* < 0.05 versus the sham group; ^#^*P* < 0.05 versus the I/R group. TNF-*α*: tumor necrosis factor alpha; IL-6: interleukin 6; IL-1*β*: interleukin1-beta.

VNS treatment greatly reduces the plasma concentrations of TNF-*α*, IL-6, and IL-1*β*. Inflammation is a key component of I/R injury. As shown in Figure 3, systemic cytokine levels were markedly increased in the I/R group (TNF-*α*: 225.43% ± 25.78% vs. 100.00% ± 14.02%; IL-6: 244.26% ± 41.26% vs. 100.00% ± 16.28%; IL-1*β*: 334.20% ± 47.96% vs. 100.00% ± 16.77%; ^∗^*P* < 0.05 for each group). Indeed, VNS produced the expected anti-inflammatory response as the serum TNF-*α*, IL-6, and IL-1*β* were markedly reduced by VNS treatment during the I/R process (TNF-*α*: 132.29% ± 14.92% vs. 225.43% ± 25.78%; IL-6: 141.68% ± 13.39% vs. 244.26% ± 41.26%; IL-1*β*: 218.98% ± 24.88% vs. 334.20% ± 47.96%; ^#^*P* < 0.05 for each group).

### 3.3. Effect of VNS on Oxidative Stress Activity in the Kidney

**Figure 4:**
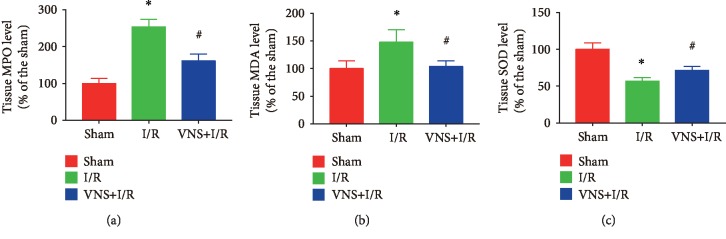
VNS attenuates oxidative stress in the kidney. (a–c) The effect of VNS on the level of MPO, MDA, and SOD in renal tissues from various groups. Data are expressed as mean ± SD with *n* = 6 per group. ^∗^*P* < 0.05 versus the sham group; ^#^*P* < 0.05 versus the I/R group. MPO: malondialdehyde; MDA: myeloperoxidase; SOD: superoxide dismutase.

As shown in Figures 4(a)–4(c), compared to the levels in the sham-operated group, the renal MDA and MPO levels were increased and SOD activity was decreased in the I/R group (MPO: 254.20% ± 20.25% vs. 100.00% ± 13.71%; MDA: 148.13% ± 22.49% vs. 100.00% ± 14.39%; SOD: 57.08% ± 4.59% vs. 100.00% ± 9.00%; ^∗^*P* < 0.05 for each group). However, VNS treatment significantly reduced renal MDA and MPO levels and increased SOD activity (MPO: 161.52% ± 18.38% vs. 254.20% ± 20.25%; MAD: 103.75% ± 10.19% vs. 148.13% ± 22.49%; SOD: 71.49% ± 5.60% vs. 57.08% ± 4.59%; ^#^*P* < 0.05 for each group), suggesting that VNS treatment alleviated oxidative stress.

### 3.4. Effect of VNS on NF-*κ*B Activation and iNOS Expression in the Kidney

**Figure 5:**
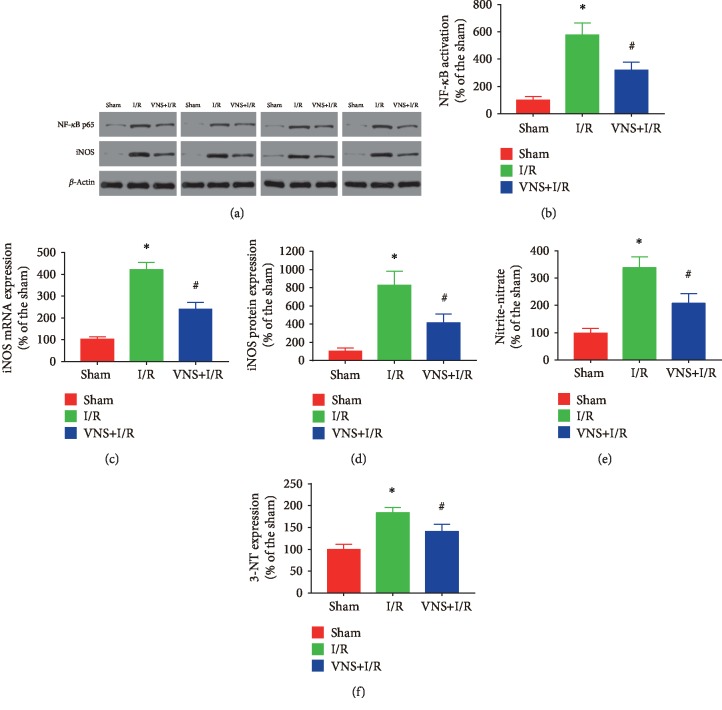
VNS decreases NF-*κ*B activation and reduces iNOS expression in the kidney. (a) Representative relative protein expression of NF-*κ*B p65 and iNOS in different groups. (b) The relative protein levels of NF-*κ*B in renal tissues are shown. (c, d) The level of iNOS mRNA and iNOS protein expression from the renal tissues in different groups. (e–f) The levels of nitrate/nitrite and 3-NT formation from the renal tissues in different groups. Data are expressed as mean ± SD with *n* = 6 per group. ^∗^*P* < 0.05 versus the sham group; ^#^*P* < 0.05 versus the I/R group. iNOS: inducible nitric oxide synthase; 3-NT: 3-nitrotyrosine.

The nuclear proteins were separated, and NF-*κ*B activity was determined. As shown in Figures 5(a)–5(d), compared to the levels in the sham-operated group, NF-*κ*B p65 protein expression was significantly higher in the renal I/R group (NF-*κ*B p65: 576.69% ± 89.04% vs. 100.00% ± 26.03%; ^∗^*P* < 0.05), which may have caused the observed increase in iNOS expression (iNOS mRNA expression: 411.38% ± 32.21% vs. 100.00% ± 11.19%; iNOS protein expression: 830.56% ± 150.12% vs. 100.00% ± 37.50%; ^∗^*P* < 0.05 for each group). Meanwhile, the renal NO metabolite levels (nitrate and nitrite levels) were significantly elevated in the rats subjected to I/R (338.00% ± 39.40% vs. 100.00% ± 18.60%; ^∗^*P* < 0.05). Importantly, VNS treatment effectively attenuated NF-*κ*B activation (NF-*κ*B p65: 320.53% ± 57.53% vs. 576.69% ± 89.04%; ^#^*P* < 0.05), attenuated the levels of renal iNOS mRNA and iNOS protein expression (iNOS mRNA: 234.14% ± 29.80% vs. 411.38% ± 32.21%; iNOS expression: 416.67% ± 94.44% vs. 830.56% ± 150.12%; ^#^*P* < 0.05 for each group), and significantly reduced NO metabolite production (207.60% ± 35.00% vs. 338.00% ± 39.40%; ^#^*P* < 0.05). Similarly, as shown in Figure 5(f), renal 3-NT formation in the kidneys was significantly enhanced in those exposed to I/R compared to that in the sham group (184.07% ± 11.40% vs. 100.00% ± 11.40%, ^∗^*P* < 0.05), and VNS treatment markedly reduced 3-NT expression (141.41% ± 16.39% vs. 184.07% ± 11.40%; ^#^*P* < 0.05). These results indicated that VNS inhibited iNOS expression induced by I/R via suppressing NF-*κ*B activation. ^∗^*P* < 0.05 versus the sham group; ^#^*P* < 0.05 versus the I/R group.

## 4. Discussion

Many studies have demonstrated that VNS treatment has a protective effect by reducing the release of different cytokines, such as TNF-*α*, IL-6, and IL-1*β*, in I/R injury [18, 19]. Inoue et al. have found that stimulation of vagal afferents or efferents 24 h before renal ischemia markedly attenuated AKI and decreased systemic inflammation depending on *α*7 nicotinic acetylcholine receptor- (*α*7nAChR-) positive splenocytes [19]. In addition, VNS has been applied to treat I/R injury-induced inflammation and oxidative stress in multiple organs [11, 16, 19]. The VNS treatment in this study effectively ameliorated renal injury caused by I/R by reducing systemic inflammation and oxidative stress. In addition, the novel findings of VNS application in this study revealed that VNS can partially restore renal function and reduce renal tubule damage via inhibiting inflammation and iNOS-mediated oxidative stress.

The autonomic nervous system plays an essential role in the control of renal function [20, 21]. In physiological states, the sympathetic nerve, mainly the renal sympathetic nerve, regulates the renin secretion rate, maintains renal vascular tone, and maintains the water and electrolyte reabsorption balance to maintain internal environment stability [22]. In AKI induced by I/R, renal blood blockade results in endothelial damage, cytokine release, oxidant stress, and autophagy. A buildup of renal toxicants can directly activate the sympathetic nerve and increase renal dysfunction and tissue damage [23, 24]. The sympathetic nerve is activated via a renocerebral sympathetic reflex, which contributes to ischemia-reperfusion-induced brain inflammation and worsening of the acute renal injury [25]. Previous study has shown NF-*κ*B activation in the Paraventricular Hypothalamic Nucleus (PVN) that contributes to sympathoexcitation [26]. Additionally, renal nerve activation is a primary mechanism driving fibrogenesis in obstructive nephropathy [27]. Recent studies have indicated that renal denervation can attenuate tubular injury, apoptosis, and renal fibrosis without altering renal function in the early period of I/R [28]. These studies indicated inhibition of sympathetic activation may suppress the NF-*κ*B activation and improve renal ischemia reperfusion. The vagal nerve is an important component of the neuroendocrine-immune axis that coordinates neural and endocrine responses to restore homeostasis in the body [29]. A previous study showed that vagal nerve activation can exert protective effects by antagonizing sympathetic activity. Meanwhile, VNS treatment for cardiac I/R injury has been tested in animal models and has been shown to act via antioxidant and anti-inflammatory mechanisms [30]. Moreover, our clinical study also revealed that noninvasive VNS improved acute myocardial injury [14]. These studies reveal that VNS may be a prospective clinical treatment for RIRI. The data from this study further indicated that VNS could prevent RIRI by inhibiting NF-*κ*B activation and antioxidant stress. Therefore, our study suggests that VNS can restore autonomic regulatory function and reduce RIRI.

The pathophysiology of AKI involves multiple mechanisms, of which oxidative stress, inflammation, and changes in gene expression activating different signaling pathways can cause severe functional impairment of the cellular membrane [31]. The functional damage of cellular components and its associated structural alterations are key triggers for acute tissue injury. In recent years, NF-*κ*B activation has been shown to be involved in experimental and human renal diseases [32]. The transcription factor NF-*κ*B controls many cellular processes, including immune and inflammatory responses, cell proliferation and migration, apoptosis, and differentiation [33]. NF-*κ*B activation can result in ROS, cytokine, and chemokine overproduction and leukocyte recruitment, which are important inflammatory triggers that initiate systemic and localized inflammatory responses [34, 35]. NF-*κ*B activation of tubular epithelial cells aggravates the systemic and intrarenal inflammation induced by I/R associated with AKI. In addition, inhibiting NF-*κ*B activation ameliorates inflammation and protects against RIRI [33]. VNS treatment can reduce inflammatory cytokine release and suppress the inflammatory response. In the current study, the VNS treatment can decrease serum TNF-*α*, IL-6, and IL-1*β* during ischemic AKI. Our data indicate that the anti-inflammatory effect of VNS might be one of the potential mechanisms by which VNS protects against RIRI.

Oxidative stress is one of the major causes contributing to RIRI. During the reperfusion phase, the immune responses are activated and the ischemic renal tissues can accumulate an abundance of reactive radicals that cause cell apoptosis and tissue damage [23, 36]. Clinical and animal studies have suggested that reducing the production of ROS can protect against RIRI. MPO is associated with the production of ROS, and SOD protects cells against injury by blocking ROS production [37]. Previous studies have shown inhibiting NF-*κ*B activation can decrease the levels of MDA and MPO and improve the activity of SOD [38, 39]. In our study, our data suggest VNS could protect the kidney against oxidative stress via inhibiting NF-*κ*B activity. iNOS has been mostly synthetized through the NF-*κ*B pathway. NF-*κ*B activation has been observed in several clinical and experimental renal ischemia models. An increase in iNOS expression results in the overproduction of NO, which rapidly reacts with superoxide anions to form another potent free radical, peroxynitrite (ONOO^−^). 3-NT, an important biomarker of ONOO^−^ formation in vivo, can cause extensive oxidation of macromolecules, directly resulting in renal dysfunction in rats [40]. In the physiological state, NO regulates vasodilation and maintains adequate glomerular function. However, upon activation of the iNOS isoform during I/R, the level of NO increases and leads to 3-NT overproduction, which directly causes cell damage [41]. In our study, our results indicate that the VNS treatment could inhibit the NF-*κ*B signaling pathway and decrease renal iNOS protein expression, plasma nitrite/nitrate levels, and renal 3-NT formation and further alleviated renal dysfunction. Therefore, VNS might exert protective effects on RIRI by inhibiting NF-*κ*B activation and iNOS-mediated oxidative stress.

Notably, our study demonstrated that VNS treatment inhibited NO production from iNOS, which in turn markedly attenuated oxidative stress and renal dysfunction. The present study indicates that VNS may serve as a potential therapeutic strategy during the I/R process.

### 4.1. Study Limitations

There are still several limitations in our study. First, we only investigated the expression of iNOS, and future studies should examine eNOS activity after renal I/R. Second, previous studies have indicated that VNS exerts protective effects via different mechanisms, and these mechanisms need to be further studied in the future. Third, the VNS frequency, intensity, and duration used here were chosen based on previous basic and clinical findings; however, the best stimulation parameters should be further explored.

## 5. Conclusion

**Figure 6:**
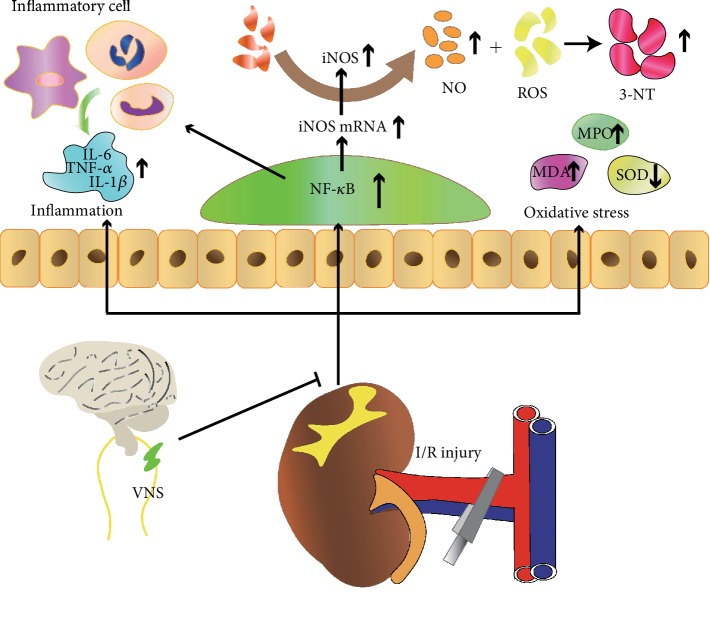
Schematic diagram depicting the protective effect of VNS on acute renal injury and the potential mechanisms.

In the present study, we observed that VNS treatment exerted a protective role in I/R-induced renal injury. The potential mechanism may be involved in inhibiting the NF-*κ*B activity, which in turn alleviated inflammation and iNOS-mediated oxidative stress (Figure 6). These results suggested that VNS may represent a novel therapeutic approach for patients with acute kidney injury.

## Data Availability

The data used to support the findings of this study are available from the corresponding authors upon request.
